# Should Mother Baby Units be renamed Parent Baby Units? A critical reflection on gendered language in perinatal psychiatry

**DOI:** 10.1177/10398562241273069

**Published:** 2024-08-19

**Authors:** Sophie Isobel

**Affiliations:** Faculty of Medicine and Health, 4334 University of Sydney, Camperdown, NSW, Australia

**Keywords:** gendered language, feminist, perinatal mental health, queer, Mother Baby Units

## Abstract

**Objective:**

Mother Baby Units provide mental health care to parents experiencing severe perinatal mental illness. The majority of admitted parents identify as mothers and are the birthing parent and primary caregiver for their infants. However, there is increasing recognition of transgender and gender diverse people who birth and parent infants, as well as awareness of the mental health needs of fathers, people in same-sex relationships, and other non-birthing parents. As such there are moves to use ungendered language for health services including renaming these units as Parent Baby Units. This paper explores this debate, critically reflecting on emergent tensions.

**Conclusion:**

Movements towards, and resistance against, changing language in perinatal mental health care are attempts to ensure the visibility of groups within mainstream services. Whether to adopt new terminology is a complex question. But ensuring MBUs meet the needs of people who require them should remain paramount.

Mother Baby Units (MBUs) are the gold standard of mental health care delivery for parents, usually mothers, experiencing severe perinatal mental illness.^1^ They are a long-fought-for addition to public mental health services in Australia. Providing acute psychiatric care to women with their infant present allows for attachment-informed mental health care and emphasises the interrelated components of perinatal mental illness, infant mental health, and relational recovery.

The majority of parents who experience perinatal mental illness are cisgender women who identify as mothers and are the birthing parent and primary caregiver for their infants. However, there is increasing recognition of transgender (trans) and people of diverse genders who birth and parent infants,^2,3^ alongside awareness of the mental health needs of fathers, people in same-sex relationships, and other non-birthing parents.^4,5^ In line with these social shifts and awareness, there are moves to use un-gendered language and rename MBUs as Parent Baby Units.

## What is in a name?

There are two MBUs in Australia that have been alternately named as a Parent Baby Unit (in New South Wales) and a Parent Infant Unit (in Victoria). The renaming of MBUs has wider implications than just the units themselves. It represents inclusivity of diverse parenting roles and parenting responsibilities, disrupting the historically mother-centric area of Western attachment research and practice. However, there are risks associated with the change, including that it may unintentionally dismiss decades-long efforts towards awareness of the unique experiences of health and illness of women, or genericise mental health care designed specifically to target the physiologic, psychologic, social and emotional impacts of early parenting for people of all genders, if any parent, regardless of attachment relationship, can be admitted if they happen to be unwell in the perinatal period.

In the context of MBUs, the shift towards using the word ‘parent’ has two distinct purposes: Inclusivity of trans and gender diverse people and people with innate variation of sex characteristics who parent; as well as inclusivity of fathers and non-birthing parents into the care environment. Despite their shared outcome, these two purposes are underpinned by differing ideologies. Accordingly, this paper aims to explore the debate about renaming units and critically reflect on emergent tensions.

## Perspectives on perinatal mental health care

Perinatal psychiatry seeks to contextualise illness in the context of pregnancy, birth, and early parenting, in recognition of the unique illness experiences that can occur in this period. Specialist services, including MBUs, developed from awareness of the differing treatment requirements during this period, as well as the importance of supporting attachment and early infant development. However, known tensions can arise between balancing the rights of women, infants, and fathers or other parents. For example, feminist scholars have criticised discourses of perinatal mental illness which emphasise the risk of the perinatal mental illness on the infant and blame women for causing adversity for infants.^6^

Feminist approaches to healthcare aim to centre women’s lived experiences, in a world primarily constructed through the eyes of men.^7^ Looking at perinatal mental health through a feminist lens outlines how patriarchal constructs and expectations of motherhood can contribute to distress and illness in the perinatal period, including through recognition of links between gender inequality and mental distress,^8^ and silencing of women’s distress. This silence can be a crucial factor in perpetuating experiences of mental illness.^9,10^ Feminist work has informed critical policies and interventions to reduce perinatal mental illness, such as mother’s groups and women’s health clinics. Thus, while decisions to rename MBUs may be framed and intended as progressive rather than political, it is important to be aware that resistance and backlash against feminist notions are often enacted through dilution, mainstreaming, and neutralising, including through calls for equality of men’s rights.

There are suggestions that fathers are overlooked within modern Western perinatal mental health care,^5^ both in the context of their partner’s mental illness as well as their own. Currently in the literature there is no mention of fathers being admitted as the primary patient in MBUs. However, the impacts of women’s MBU admissions on their partners has been well explored, with calls for greater inclusion in care due to fathers’ experiences of overwhelm, frustration, helplessness, fear, isolation, and distress.^11,12^ One indication for changing language is to more overtly signal recognition of partners and their roles in parenting. However, this should occur alongside consideration of what it also means in practice for women and women’s services to shift towards inclusive care: How does treatment differ if fathers or non-birthing parents are admitted or actively included in all aspects of care? Across perinatal services gendered language is an outcome of important historical efforts towards recognising the gendered experiences of pregnancy and birth and perinatal health, as well as midwifery-led efforts to safeguard the birthing process from patriarchal control.^13^ These acts do not intend to exclude men or non-birthing parents, yet it is important that ongoing due diligence to women and other birthing parents’ experiences is enacted, alongside progressive change.

Renaming MBUs may inadvertently agitate tensions between Feminist, Trans and Queer movements by tapping into complex intersections around issues of discourse, power, and bodies, as well as those of gender and sexuality. For example, the use of gender-specific language such as ‘women’ and ‘mothers’ can contribute to the erasure of trans and gender diverse people who are pregnant.^14^ Renaming units aligns to wider pushes across perinatal services, to remove gender-specific terms such as ‘women’ and ‘mothers’ from discussions of pregnancy and birth to signal acknowledgement of the needs of people who may be pregnant or birth infants but do not identify as women and for inclusivity of trans and gender diverse parents, and those in same-sex relationships.^15^ Trans and gender diverse people, as well as people of diverse sexualities are known to experience high levels of discrimination that limit their access to healthcare in general,^16,17^ with institutional cis-heteronormativity and cis-genderism, or the assumptions that everyone identifies as the gender they were assigned at birth and identify as heterosexual, particularly pervasive across pregnancy, birth and parenting.^2,3^ Efforts towards altering cis-heteronormative structures disrupt discourses that position such assumptions as ‘normal’,^7^ while realigning services to meet the needs of the diverse populations who access them.^18^ This is particularly important in areas where people of diverse genders or sexuality may be excluded through service nomenclature.

## Language as a powerful force

It is a continuing challenge for all services to ongoingly identify and use language that is clear, accurate and dignity-preserving for all people who access care.^15^ Opposing, and at times contradicting, reasons for renaming MBUs, highlight immediate tensions in the shared outcome of changing language to be non-gender specific for the purposes of including fathers or alternately, disrupting cis-heteronormative assumptions. Although framed differently, both movements towards, and resistance against, changing language are attempts to resist systems that can make experiences invisible – whether they be those of women or fathers or people who are trans or of diverse genders. Thus, while gender-neutral language is encouraged, critical reflection on the implications of using it meaningfully is needed, with attention to alternate ways to demonstrate inclusivity also required if current linguistic options are limited.

Language reflects conventions of culture and mainstream patterns of thought, while also shaping people’s understanding of the world.^19^ Language can also reinforce cultural assumptions, such as who is considered a parent, and which roles are of primary perinatal importance. Language is constantly evolving and often imperfect. One challenge in renaming MBUs for inclusivity, is a lack of consensus of which terms are better. Many people who give birth but do not identify as women use differing terms to refer to their gender identity.^20^ Subsequently a variety of attempts have been made to avoid gendered language, including through focussing on social roles, for example, ‘parents’ and ‘families’, as well as a focus on actions or body parts, for example, ‘people who birth’ or ‘people with wombs’.^15^ Each of these is inclusive in some ways and marginalising or excluding in others. There are also calls for additive language^21^ such as ‘mothers and birthing parents’. While additive language is at times a workable compromise, it can also inadvertently ‘other’ those who do not fit the implied norm and potentially lead to dissonance between language and practice. For example, if only parents who have given birth are admitted to MBUs then ‘mother and birthing parents’ may be appropriate, but if fathers, adoptive parents, or non-birthing same-sex parents can also be admitted, additive language needs to include this also.

Changing language can be crucial for social change. Yet, it can lead to lack of clarity, misunderstandings and reduced visibility of groups.^15^ At times it can also result in unexpected complexities in service delivery.^15^ For example, gender-inclusive language can lead to confusion about the purpose of MBUs, positioning them as early parenting services or family support services rather than acute psychiatric units, and creating uncertainty about admitting non-traditional family groups (such as polyamorous parents). Thus, the need for ongoing sensitive consultation with diverse groups is required.

Concurrent to recognition that gender itself is complex, comes complexity in how to best represent and talk about gender, with awareness that many attempts may result in some marginalisation. Resistance against gendered language it not uncommon and can be used as an intentional distraction against causes. However, moving to gender-neutral language also commonly makes people uncomfortable, particularly those adequately addressed through existing terms. Thus, whether MBUs should be known as Mother or Parent Baby Units will be an ongoing debate with compelling arguments both ways and innovative solutions ahead, amid shared purposes of protecting the rights of cisgender women and others experiencing severe perinatal mental illness.

While disagreement about language can be generative, it can also distract from commonalities and force requirements to take sides^13^ where instead, attention could be focused on how to be inclusive of experiences of severe perinatal mental illness, regardless of gender or sex. Importantly for MBUs, if gender-neutral language is adopted without congruent inclusive environments and practices, there is a risk of tokenism and dissonance between nomenclature and actions. Across sectors, services are working to blend language to be inclusive and supportive of all people without being exclusionary, inaccurate or harmful, while ongoing language evolution occurs.^22^ In the meantime, creative and collaborative solution-generation is needed, alongside tolerance and flexibility.

## Conclusion

The question of whether Mother Baby Units should change their name to Parent Baby Units requires reflection on the services they aim to provide. A focus on care provision (e.g. ‘Perinatal Mental Health Units’) rather than explicitly naming the recipients of care could reduce focus on the gender identity of people admitted, while solutions to broader issues of language and gender evolve. Regardless of unit names, it is important for services to recognise the diverse genders of people who birth, parent, and experience perinatal mental illness, while also not neutralising the experiences of a population, largely women, who have been historically silenced. Paradoxically, while inclusivity occurs through shifting language, so does erasure, with any selection of language reinforcing values and marginalising concurrently.

Language systems are evolving to catch up with social change and it is not uncommon for language to change slower than social constructions of experience. Whether to adopt new terminology is a complex question, and critical reflection by people who access services, clinicians and others is needed. There will undoubtedly be ongoing change in the use of gendered language across society, including healthcare, and there is a need for practice and service models to also evolve alongside language, to ensure inclusivity of diversity. In the meantime, ensuring MBUs meet the needs of people who require them should remain paramount.
